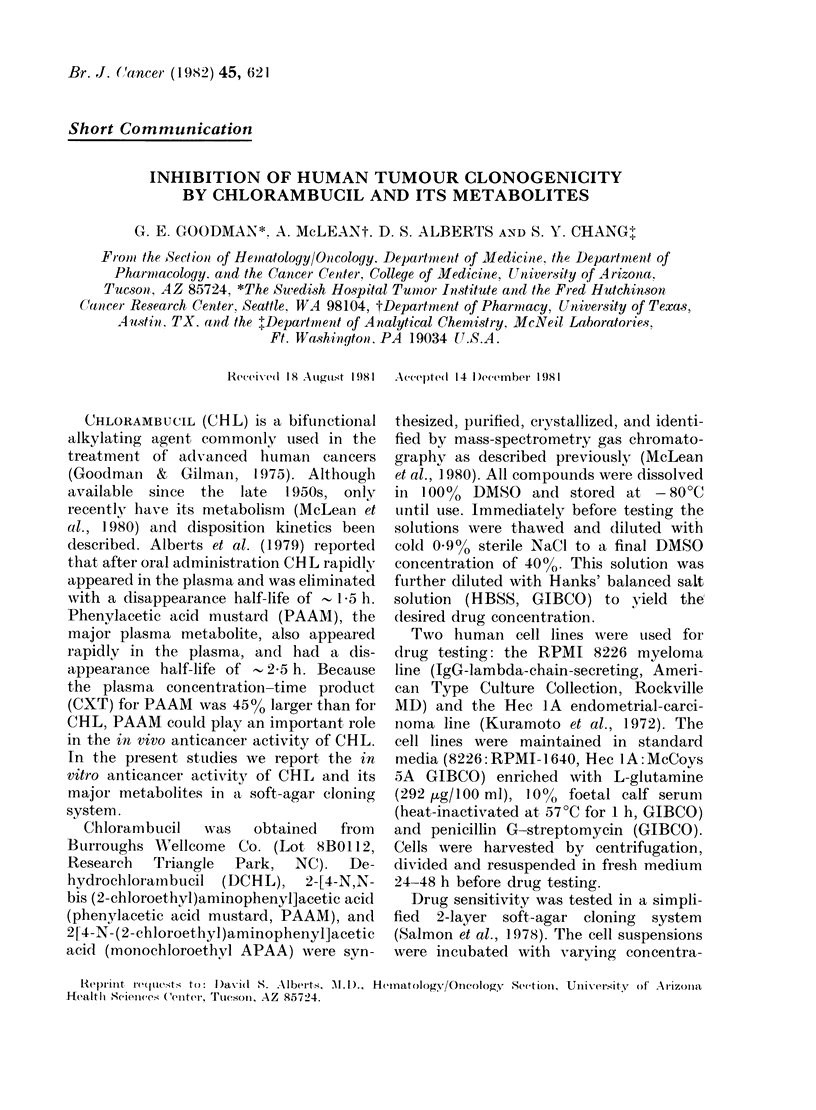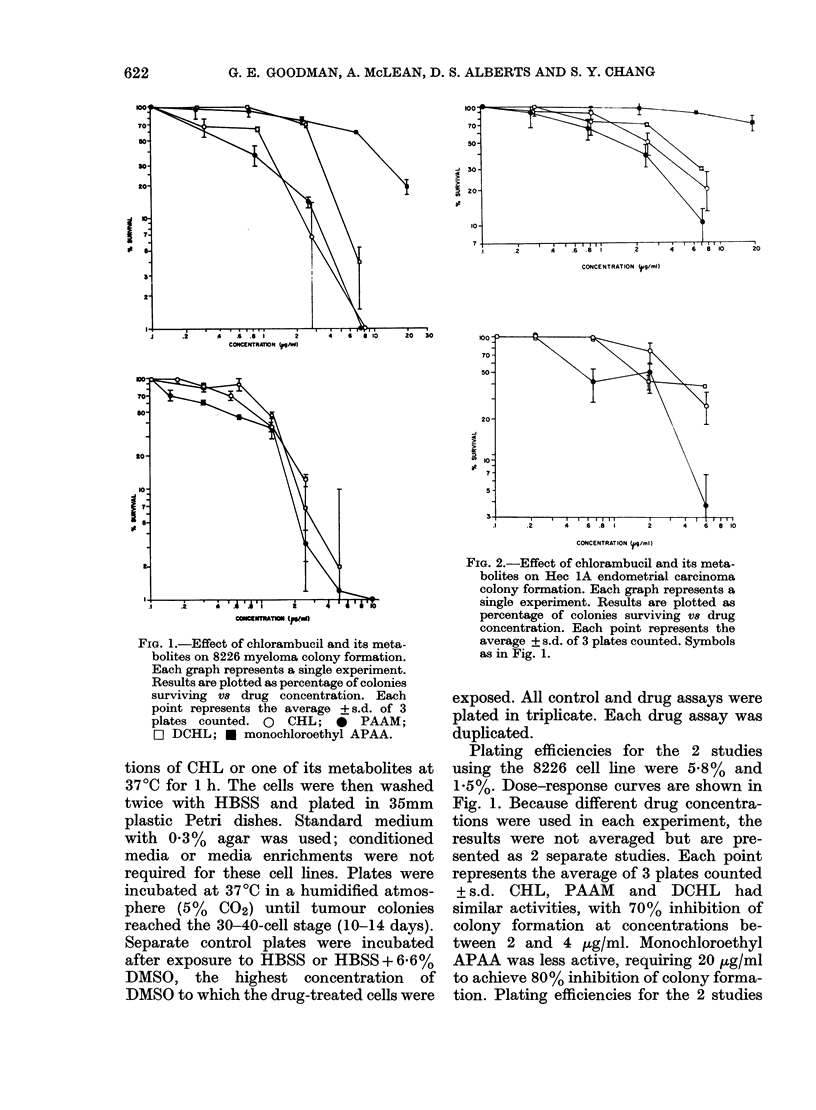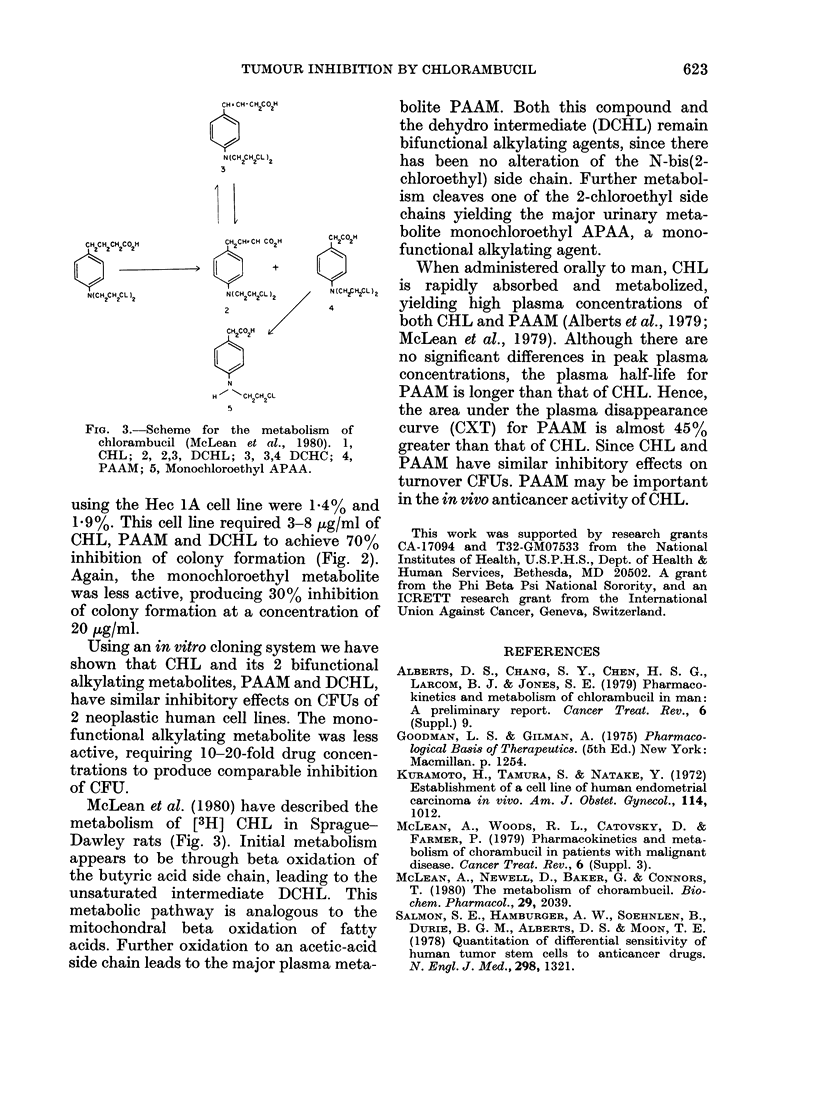# Inhibition of human tumour clonogenicity by chlorambucil and its metabolites.

**DOI:** 10.1038/bjc.1982.101

**Published:** 1982-04

**Authors:** G. E. Goodman, A. McLean, D. S. Alberts, S. Y. Chang


*Br. J. Cancer* (1982) **45**, 621

***Short Communication***

### INHIBITION OF HUMAN TUMOUR CLONOGENICITY BY CHLORAMBUCIL AND ITS METABOLITES

G. E. GOODMAN*, A. McLEAN†, D. S. ALBERTS AND S. Y. CHANG‡

*From the Section of Hematology/Oncology, Department of Medicine, the Department of Pharmacology, and the Cancer Center, College of Medicine, University of Arizona, Tucson, AZ* 85724, **The Swedish Hospital Tumor Institute and the Fred Hutchinson Cancer Research Center, Seattle, WA* 98104, *†Department of Pharmacy, University of Texas, Austin, TX, and the ‡Department of Analytical Chemistry, McNeil Laboratories, Ft. Washington, PA* 19034 *U.S.A.*

Received 18 August 1981 Accepted 14 December 1981

CHLORAMBUCIL (CHL) is a bifunctional alkylating agent commonly used in the treatment of advanced human cancers (Goodman & Gilman, 1975). Although available since the late 1950s, only recently have its metabolism (McLean *et al.*, 1980) and disposition kinetics been described. Alberts *et al.* (1979) reported that after oral administration CHL rapidly appeared in the plasma and was eliminated with a disappearance half-life of ~1·5 h. Phenylacetic acid mustard (PAAM), the major plasma metabolite, also appeared rapidly in the plasma, and had a disappearance half-life of ~2·5 h. Because the plasma concentration–time product (CXT) for PAAM was 45% larger than for CHL, PAAM could play an important role in the *in vivo* anticancer activity of CHL. In the present studies we report the *in vitro* anticancer activity of CHL and its major metabolites in a soft-agar cloning system.

Chlorambucil was obtained from Burroughs Wellcome Co. (Lot 8B0112, Research Triangle Park, NC). Dehydrochlorambucil (DCHL), 2-[4-N,N-bis (2-chloroethyl)aminophenyl]acetic acid (phenylacetic acid mustard, PAAM), and 2[4-N-(2-chloroethyl)aminophenyl]acetic acid (monochloroethyl APAA) were synthesized, purified, crystallized, and identified by mass-spectrometry gas chromatography as described previously (McLean *et al.*, 1980). All compounds were dissolved in 100% DMSO and stored at −80°C until use. Immediately before testing the solutions were thawed and diluted with cold 0·9% sterile NaCl to a final DMSO concentration of 40%. This solution was further diluted with Hanks' balanced salt solution (HBSS, GIBCO) to yield the desired drug concentration.

Two human cell lines were used for drug testing: the RPMI 8226 myeloma line (IgG-lambda-chain-secreting, American Type Culture Collection, Rockville MD) and the Hec 1A endometrial-carcinoma line (Kuramoto *et al.*, 1972). The cell lines were maintained in standard media (8226:RPMI-1640, Hec 1A:McCoys 5A GIBCO) enriched with L-glutamine (292 μg/100 ml), 10% foetal calf serum (heat-inactivated at 57°C for 1 h, GIBCO) and penicillin G–streptomycin (GIBCO). Cells were harvested by centrifugation, divided and resuspended in fresh medium 24–48 h before drug testing.

Drug sensitivity was tested in a simplified 2-layer soft-agar cloning system (Salmon *et al.*, 1978). The cell suspensions were incubated with varying concentra-

Reprint requests to: David S. Alberts, M.D., Hematology/Oncology Section, University of Arizona Health Sciences Center, Tucson, AZ 85724.

FIG. 1.—Effect of chlorambucil and its metabolites on 8226 myeloma colony formation. Each graph represents a single experiment. Results are plotted as percentage of colonies surviving *vs* drug concentration. Each point represents the average ± s.d. of 3 plates counted. ○ CHL; ● PAAM; □ DCHL; ■ monochloroethyl APAA.

FIG. 2.—Effect of chlorambucil and its metabolites on Hec 1A endometrial carcinoma colony formation. Each graph represents a single experiment. Results are plotted as percentage of colonies surviving *vs* drug concentration. Each point represents the average ± s.d. of 3 plates counted. Symbols as in Fig. 1.

tions of CHL or one of its metabolites at 37°C for 1 h. The cells were then washed twice with HBSS and plated in 35mm plastic Petri dishes. Standard medium with 0·3% agar was used; conditioned media or media enrichments were not required for these cell lines. Plates were incubated at 37°C in a humidified atmosphere (5% $CO_2$) until tumour colonies reached the 30–40-cell stage (10–14 days). Separate control plates were incubated after exposure to HBSS or HBSS + 6·6% DMSO, the highest concentration of DMSO to which the drug-treated cells were exposed. All control and drug assays were plated in triplicate. Each drug assay was duplicated.

Plating efficiencies for the 2 studies using the 8226 cell line were 5·8% and 1·5%. Dose–response curves are shown in Fig. 1. Because different drug concentrations were used in each experiment, the results were not averaged but are presented as 2 separate studies. Each point represents the average of 3 plates counted ± s.d. CHL, PAAM and DCHL had similar activities, with 70% inhibition of colony formation at concentrations between 2 and 4 µg/ml. Monochloroethyl APAA was less active, requiring 20 µg/ml to achieve 80% inhibition of colony formation. Plating efficiencies for the 2 studies

FIG. 3.—Scheme for the metabolism of chlorambucil (McLean *et al.*, 1980). 1, CHL; 2, 2,3, DCHL; 3, 3,4 DCHC; 4, PAAM; 5, Monochloroethyl APAA.

using the Hec 1A cell line were 1·4% and 1·9%. This cell line required 3–8 μg/ml of CHL, PAAM and DCHL to achieve 70% inhibition of colony formation (Fig. 2). Again, the monochloroethyl metabolite was less active, producing 30% inhibition of colony formation at a concentration of 20 μg/ml.

Using an *in vitro* cloning system we have shown that CHL and its 2 bifunctional alkylating metabolites, PAAM and DCHL, have similar inhibitory effects on CFUs of 2 neoplastic human cell lines. The monofunctional alkylating metabolite was less active, requiring 10–20-fold drug concentrations to produce comparable inhibition of CFU.

McLean *et al.* (1980) have described the metabolism of [$^3$H] CHL in Sprague–Dawley rats (Fig. 3). Initial metabolism appears to be through beta oxidation of the butyric acid side chain, leading to the unsaturated intermediate DCHL. This metabolic pathway is analogous to the mitochondral beta oxidation of fatty acids. Further oxidation to an acetic-acid side chain leads to the major plasma metabolite PAAM. Both this compound and the dehydro intermediate (DCHL) remain bifunctional alkylating agents, since there has been no alteration of the N-bis(2-chloroethyl) side chain. Further metabolism cleaves one of the 2-chloroethyl side chains yielding the major urinary metabolite monochloroethyl APAA, a monofunctional alkylating agent.

When administered orally to man, CHL is rapidly absorbed and metabolized, yielding high plasma concentrations of both CHL and PAAM (Alberts *et al.*, 1979; McLean *et al.*, 1979). Although there are no significant differences in peak plasma concentrations, the plasma half-life for PAAM is longer than that of CHL. Hence, the area under the plasma disappearance curve (CXT) for PAAM is almost 45% greater than that of CHL. Since CHL and PAAM have similar inhibitory effects on turnover CFUs. PAAM may be important in the *in vivo* anticancer activity of CHL.

This work was supported by research grants CA-17094 and T32-GM07533 from the National Institutes of Health, U.S.P.H.S., Dept. of Health & Human Services, Bethesda, MD 20502. A grant from the Phi Beta Psi National Sorority, and an ICRETT research grant from the International Union Against Cancer, Geneva, Switzerland.

### REFERENCES

ALBERTS, D. S., CHANG, S. Y., CHEN, H. S. G., LARCOM, B. J. & JONES, S. E. (1979) Pharmacokinetics and metabolism of chlorambucil in man: A preliminary report. *Cancer Treat. Rev.*, **6** (Suppl.) 9.

GOODMAN, L. S. & GILMAN, A. (1975) *Pharmacological Basis of Therapeutics.* (5th Ed.) New York: Macmillan. p. 1254.

KURAMOTO, H., TAMURA, S. & NATAKE, Y. (1972) Establishment of a cell line of human endometrial carcinoma *in vivo*. *Am. J. Obstet. Gynecol.*, **114**, 1012.

MCLEAN, A., WOODS, R. L., CATOVSKY, D. & FARMER, P. (1979) Pharmacokinetics and metabolism of chorambucil in patients with malignant disease. *Cancer Treat. Rev.*, **6** (Suppl. 3).

MCLEAN, A., NEWELL, D., BAKER, G. & CONNORS, T. (1980) The metabolism of chorambucil. *Biochem. Pharmacol.*, **29**, 2039.

SALMON, S. E., HAMBURGER, A. W., SOEHNLEN, B., DURIE, B. G. M., ALBERTS, D. S. & MOON, T. E. (1978) Quantitation of differential sensitivity of human tumor stem cells to anticancer drugs. *N. Engl. J. Med.*, **298**, 1321.